# Outcomes in High Perianal Fistula Repair Using Video-Assisted Anal Fistula Treatment Compared With Seton Use: A Randomized Controlled Trial

**DOI:** 10.7759/cureus.22166

**Published:** 2022-02-13

**Authors:** Sumera Siddique, Shabbar H Changazi, Samiullah Bhatti, Barza Afzal, Zulqarnain Hyidar, Aveena Rehman, Qamar Ashfaq Ahmad, Mahmood Ayyaz

**Affiliations:** 1 Department of General Surgery, Services Hospital Lahore, Lahore, PAK; 2 Department of Surgery, Services Hospital Lahore, Lahore, PAK; 3 Department of Surgery, Services Institute of Medical Services, Lahore, Lahore, PAK

**Keywords:** anal incontinence, recurrence, high perianal fistula, seton, vaaft

## Abstract

Background

Anal fistula, or fistula-in-ano, is a chronic abnormal communication between the epithelialized surface of the anal canal and the perianal skin. Video-assisted anal fistula treatment (VAAFT) is a novel, minimally invasive, and sphincter-saving alternative to traditional seton use. This study aimed to determine the short-term and long-term outcomes of VAAFT compared with seton treatment.

Material and methods

This randomized control trial was conducted at the Department of Surgery, Services Hospital, Lahore, from August 2014 to July 2020. Patients were randomly assigned to either the VAAFT group or the seton group, and postoperative outcomes were assessed for up to three years.

Results

The study included 80 patients (64 men and 16 women) with a mean age of 39.1 ± 11.2 years. The most common type of fistula was a transsphincteric fistula (n=36, 45%). The mean duration of surgery was significantly longer in the VAAFT group (78.6 minutes) compared with the seton group (36.97 minutes; p=0.000). The mean pain score was significantly higher in the VAAFT group (4.22) compared to the seton group (2.82, p=0.000). The mean time to return to work was shorter in the VAAFT group (7.4 days) than in the seton group (9.2 days, p=0.000). The mean healing time was significantly shorter for patients treated with VAAFT (5.75 weeks) than for those treated with a seton (9.7 weeks; p=0.000). Fistula recurrence after one, two, or three years was not significantly different between groups, and neither group had incidences of anal incontinence.

Conclusions

VAAFT is associated with earlier healing time and earlier return to work than the traditional seton technique, with no significant difference in fistula recurrence. VAAFT is minimally invasive and, when used in patients where indicated, allows for a prompter return to routine life for the patients, which is an optimal outcome for both patients and physicians.

## Introduction

Anal fistula is the most common perianal disease, with an incidence of nine in 100,000 patients. Anal fistula classification is based on the lesion’s location above or below the dentate line, defining a high or low anal fistula. Formation of a fistula tract following the anorectal abscess occurs in up to 40% of cases [1]. There are typically eight to ten anal crypt glands at the level of the dentate line in the anal canal, arranged circumferentially. These glands provide a path for infectious agents to reach the intramuscular spaces. The cryptoglandular hypothesis states that infection begins in the anal canal glands and progresses into the muscular wall of the anal sphincters to cause an anorectal abscess [2].

Various classifications have been proposed according to the internal opening; however, the standard remains Park’s classification. There are four types of fistula-in-ano in Park’s classification: (1) intersphincteric (i.e., the fistula spans 70% of the area between internal and external sphincters), (2) transsphincteric (i.e., the fistula spans 25% of the external sphincter), (3) suprasphincteric (i.e., the fistula is above the sphincters), and (4) extrasphincteric (i.e., the fistula is above and through the levator ani) [2].

A high anal fistula is difficult to treat because of its location. They are typically diagnosed via endoluminal ultrasound and magnetic resonance imaging, and they are typically treated with seton placement. However, these treatment methods are associated with fecal incontinence, fistula recurrence, and prolonged, painful healing. Other methods have been devised to mitigate these complications, such as ligation of the intersphincteric fistula tract, glue repair, and flap advancement. Another recently introduced method, video-assisted anal fistula treatment (VAAFT), proposed by Meinero, has fewer complications [3]. According to Meinero, VAAFT was associated with recurrence in 26 out of 136 patients (26.5%). Ege et al. found that patients treated with a seton had a mean pain score of 3.23, and recurrence was noted in only two of 147 patients (1.5%) [4-7].

The purpose of this prospective study is to compare the postoperative outcomes of VAAFT with traditional seton repair in the management of high perianal fistula by assessing postoperative recurrence.

## Materials and methods

This randomized controlled trial was conducted at the Department of Surgery, Services Hospital, Lahore, from August 2014 to July 2020. Ethics approval was obtained from the institutional review board of the Services Institute of Medical Sciences, Lahore, Pakistan. Patients of both sexes with an average age ranging from 18 to 65 years with high perianal fistula were included in the study. High perianal fistulas were defined as intersphincteric fistulas with more than 50% of internal anal sphincter involvement, transsphincteric fistulas with more than 50% of sphincter complex involvement, extrasphincteric fistulas, or suprasphincteric fistulas. The anatomy of fistulas was delineated by three-dimensional endoanal ultrasound. Patients with suspected malignancy, as determined by the presence of a mass on digital rectal examination, a history of previous perianal surgery, a medical record-based history of inflammatory bowel disease, or uncontrolled diabetes were excluded from the study. After receiving written permission from the ethical review committee, patients fulfilling the inclusion criteria were admitted through the outpatient department. Informed consent was obtained from all the patients included in the study.

Patients were randomly assigned to either repair via VAAFT or traditional seton use via a computer-generated random number. Patients assigned to the VAAFT group received the following procedure. The external opening was widened with a probe, and a fistulascope was inserted to delineate the primary and secondary tracts and locate the internal opening. The internal opening was then stitched with Vicryl™ (polyglactin 910) 2-0 suture (Ethicon Inc., Raritan, New Jersey, USA) through the anal route with the help of a proctoscope. The tract of the fistula was washed and debrided through the scope and cauterized. Finally, the external opening was excised and sent for biopsy. For the other treatment group, a seton was applied at this stage. With a 10-cc syringe, hydrogen peroxide was applied to the external opening, and the internal opening was located by direct visualization of the anal canal with a proctoscope. A probe was inserted into the external opening and carefully maneuvered through the internal opening. Silk 1/0 suture was then tied to the tip of the probe, which was then squeezed out of the external opening. The suture was then tied around the sphincter and through the fistula tract. Later, the seton was tightened at four-week intervals under local anesthesia until the suture cut through the sphincter. All surgeries were performed by trained consultants under general anesthesia. Intravenous ketorolac 90 mg/day and intravenous acetaminophen 3 g/day were used as postoperative anesthesia for both groups.

Postoperative pain was determined 12 hours after the surgery via the visual analog pain score. Patients were then monitored via follow-up in the outpatient clinic at two and four weeks, three and six months, and one, two, and three years to determine when a patient could return to work, total healing time, and monitor for recurrence (primary outcome) and anal incontinence. Healing of the fistula was assessed clinically by the consultant surgeon and was defined as complete closure of the external opening (perianal wound) without any complaints of discharge or pus from the scar. Anal continence was assessed subjectively by asking the patient whether he had episodes of involuntary passage of flatus or stools on each follow-up. All the data were recorded on a predesigned proforma. The data were analyzed using IBM SPSS Statistics for Windows, Version 21.0 (IBM Corp., Armonk, NY, USA). The categorical variables like sex, type of fistula, and recurrence were presented as frequencies and percentages. In contrast, continuous variables like age, duration of surgery, pain score, the time required to return to work, and healing time were presented as the mean standard deviation. The mean pain score, mean duration of surgery, mean time in days to return to work, and mean time of healing were compared using a t-test. Recurrence was compared using a chi-square test. Differences were considered statistically significant at p ≤ 0.05. A sample size of 76 patients was calculated (n=38 in the VAAFT group and n=38 in the seton group) at 80% power of the study and 95% confidence interval, taking proportionately an expected percentage of 26.55% and 1.5% of recurrence in the VAAFT and seton groups in 12-month follow-up, respectively. However, for convenience, a sample of 80 patients (n=40 in the VAAFT group and n=40 in the seton group) was adopted, and we used nonprobability consecutive sampling.

## Results

A total of 84 participants were recruited during initial screening from August 1, 2014 to July 31, 2020. Two patients were diagnosed with Crohn’s disease and were excluded before the surgery. Two patients were diagnosed with squamous cell carcinoma of the anal canal on biopsy after surgery and were excluded from surgery. Therefore, 80 patients were included in the surgery and monitored via follow-up for three years after the surgery, and all patients completed the study. 

The study included 80 patients (64 men and 16 women) with a mean age of 39.1 ± 11.2 years. The most common type of fistula was a transsphincteric fistula (n=36; 45%) followed by an intersphincteric fistula (n=31; 38.5%; Table 1).

**Table 1:** Baseline characteristics of patients. VAAFT: video-assisted anal fistula treatment.

		VAAFT	Seton	p-value
Age (years)		39.9 ± 12.4	38.3 ± 10.0	0.548
Gender	Male	33 (82.5%)	31 (77.5%)	0.576
	Female	7 (17.5%)	9 (22.5%)	
	Total	40 (100%)	40 (100%)	
Type of fistula	Intersphincteric	14 (35%)	17 (42.5%)	0.843
	Transsphincteric	20 (50%)	16 (40%)	
	Suprasphincteric	5 (12.5%)	6 (15%)	
	Extrasphincteric	1 (2.5%)	1 (2.5%)	
	Total	40 (100%)	40 (100%)	

The mean duration of surgery in VAAFT patients was 78.60 minutes; seton patients had a mean duration of surgery of 36.97 minutes (p=0.000). The mean pain score was statistically higher in VAAFT patients (4.22) than in patients treated using a seton (2.82; p=0.000). The mean time required to return to work was significantly shorter in VAAFT patients (7.42 days) than in seton patients (9.27 days; p=0.000). The mean healing time was significantly shorter in patients receiving VAAFT (5.75 weeks) compared to patients treated using a traditional seton (9.7 weeks; p=0.000; Table 2).

**Table 2:** Comparison of short-term outcomes. VAAFT: video-assisted anal fistula treatment.

	VAAFT	Seton	p-value
Duration of surgery (min)	78.60 ± 26.24	36.97 ± 12.98	0.000
Pain score	4.22 ± 1.83	2.82 ± 1.58	0.000
Return to work (days)	7.42 ± 1.78	9.27 ± 2.06	0.000
Healing time (weeks)	5.75 ± 1.17	9.7 ± 1.87	0.000

At one, two, and three years, the fistula recurrence rate was higher in VAAFT patients than in seton, but the difference was not statistically significant (Table 3).

**Table 3:** Comparison of recurrence rates. VAAFT: video-assisted anal fistula treatment.

	VAAFT	Seton	p-value
One year, n (%)	6 (15%)	5 (12.5%)	0.745
Two years, n (%)	8 (20%)	5 (12.5%)	0.363
Three years, n (%)	10 (25%)	5 (12.5%)	0.152

We found no anal incontinence in either test group.

## Discussion

In this study, the mean duration of surgery was significantly shorter in the seton group than in the VAAFT group, and the mean pain score was significantly lower in the seton group than in the VAAFT group. However, the mean time required to return to work and the mean healing time were significantly shorter in VAAFT patients than in seton patients, and recurrence rates were not significantly different between groups. Only one other trial compared VAAFT and seton use in treating perianal fistula. Zheng et al. [8] demonstrated that the mean operative time was shorter in VAAFT than in seton-use, and the mean pain score was lower in VAAFT compared to seton use, which does not align with our results. The discrepancy in these results may be due to differing levels of familiarity with the procedure of VAAFT, which was developed in our clinic several years ago. Also, we used silk 1/0 suture for the seton in our study. The Zheng study also found no difference in recurrence rates between the two procedures, which does align with our findings.

In our study, patients receiving repair using a seton had a three-year recurrence rate of 12.5%, which was higher than several other studies. Choi et al.’s 2010 prospective trial used a loose seton for high perianal fistula repair, and they only had a single case with a recurrence (8%) [9]. Chuang-Wei et al., in 2008, with an elastic band seton fistula repair, also reported a recurrence of perianal fistula in one patient (0.9%) [10]. Ege et al. found that patients treated with a seton had complete healing at three months in 100% of cases, while recurrence was noted in two patients (1.5%; one at six months, one at 12 months) [5]. Munir et al. described a recurrence rate of 3.3% after seton placement for high perianal fistulas [11]. In each of these studies, the follow-up period was not more than six to 12 months, and our patients were monitored via follow-up for up to three years, which might explain the higher recurrence in our study. Another significant finding of this study was that there was no incidence of anal incontinence in the seton group, which was in concordance with a study conducted by Andreou et al [12]. Other studies showed a rate of anal incontinence ranging from 9% to 20%, which was significantly higher than our results. The reason might be the technique of seton placement or the material used as seton (silk 1/0 in our case).

In VAAFT, the rate of recurrence was 25% at three years’ follow-up. According to Meinero, in VAAFT, recurrence was noted in 26 of 136 patients (26.5%) at 12 months of follow-up [4]. Our recurrence rate is much lower than that reported by Meinero. However, other studies showed the recurrence rate as much lower than ours, such as a study by Mendes et al. that reported that after a five-month follow-up of eight patients with perianal fistula, only one patient had a recurrence (12.5%) [13]. Walega et al. reported a 17% recurrence of perianal fistula in 18 patients who underwent surgery via VAAFT [14]. Kochhar et al. reported a recurrence rate of 15.85% with VAAFT during a six-month follow-up [15].

In our study, the five seton patients and five of the ten VAAFT patients who developed recurrence had low perianal fistulas for which fistulectomy was performed. The remaining five VAAFT patients who developed recurrence had a high perianal fistula, which was treated with seton placement.

Our study was limited in that it was a single-center study, which prohibits generalizing our results to a wider population. Also, the procedure we used did not inject glue into the remaining tract following VAAFT (as advised by the inventor of this procedure, and we do not know whether this procedural alteration will affect fistula recurrence rates). Our study was not free of bias in that we have more than 10 years of experience with placing setons in fistula repair, and VAAFT is a relatively new modality with which we have less experience. It remains to be seen whether additional experience with VAAFT will affect fistula recurrence rates compared to seton use. Finally, we measured anal incontinence subjectively: the patient knew which group he/she was in, and had to indicate whether he/she involuntary passed flatus or stools. This may be another potential bias in this trial. It would have been better if we had measured anal incontinence objectively through a scoring system or investigations such as anorectal manometry, endoanal ultrasonography, or magnetic resonance.

Few studies exist to compare VAAFT with seton use, and further research may have a long-lasting and positive impact on the management of complex fistulas, with the potential to altogether change their management. Our study further explores complex fistula management and serves as a guide for other surgeons in treating their patients effectively. Given the promising nature of our results, we strongly encourage further research into this new modality.

## Conclusions

In conclusion, VAAFT is an innovative sphincter-saving surgery performed for the treatment of high perianal fistulas. Irrespective of cost, VAAFT is associated with earlier healing time and a return to work compared with traditional seton use, with a similar recurrence rate between the two procedures over three years. The reduced healing time and shorter return to work associated with VAAFT have the potential to improve hospital patient burden, patient quality of life, and, ultimately, the national economy. Larger multi-center trials are warranted to explore this potential benefit to the broader community.
